# Collectanea

**Published:** 1829-09

**Authors:** 


### COLLECTANEA.

Floriferis ut apes in saltibus omnia libant,
Omnia nos, itidem, depascimur aurea dicta.

### ANATOMY.

Dr. Weber *on the Skin.*—Dr. Weber is professor of anatomy at Leipzig. In opposition to the opinion of Dr. Eichorn, whose observations were cited in a former Number of this Journal, Dr. W. asserts that the sebaceous follicles of the skin are organs distinct from the bulbs of the hair, and that they exist over the whole surface, excepting the palms of the hands and soles of the feet. The bulbs of the large hair (*gros poils*) are situated very deeply in the derm, and sometimes penetrate even into the subcutaneous adipose tissue: the sebaceous follicles, on the contrary, are nearer to the cutaneous surface, and are never found extending to the adipose structure. Their size, also, says he, is too large to permit them to be confounded with the bulbs of hair, which are much smaller. In new-born children, sebaceous follicles may be discovered on all parts of the skin, with the two exceptions already named. The skin of the scrotum shows them very much developed: each of these follicles is composed of four or five compartments, or cells, agglomerated together; their transverse diameter exceeds their depth. The greatest diameter observed by the author was a quarter of a line.

Dr. Weber also examined the hair of the body, in order to ascertain whether it is hollow, but he found no canal or any cellular structure within it. Neither is this hair cylindrical: it is rather elliptical or oval, like the negro hair, in which the flattened form seems necessary to admit of the curl or twist.—*North American Med. and Surg. Journal.*

M. Breschet *on a Cavity and Fluid in the Membrana Caduca.*—M. B. thinks he has found, in the membrana caduca, an undescribed fluid, and which perhaps performs an important part in the nutrition of the fœtus. This fluid should exist in greater quantity in proportion as the allantois and vesicula umbilicalis are smaller. The tomentose portion of the chorion might derive from it the materials of nutriment for the embryo. The cavity of the caduca and its liquid remain even until after the third month of gestation.—*Bulletin.*

### PHYSIOLOGY.

*Influence of the Male extending beyond a single Impregnation.*—A seven-eighths Arabian mare, belonging to the Earl of Morton, which had never been bred from before, had a mule by a quagga: subsequently she had three foals by a black Arabian horse. The two first of these are thus described: "They have the character of the Arabian breed as decidedly as can be expected where fifteen-sixteenths of the blood are Arabian, and they are fine specimens of that breed; but, both in their colour and in the hair of their manes, they have a striking resemblance to the quagga. Their colour is bay, marked more or less like the quagga in a darker tint. Both are distinguished by the dark line along the ridge of the back, the dark stripes across the forehand, and the dark bars across the back part of the legs. Both their manes are black: that of the filly is short, stiff, and stands upright; that of the colt is long, but so stiff as to arch upwards, and to hang clear of the sides of the neck; in which circumstance it resembles that of the hybrid. This is the more remarkable as the manes of the Arabian breed hang lank and closer to the neck than those of most others."* Mr. Mayo, who quotes this curious fact in his excellent "Outlines of Physiology," observes, that "a similar occurrence to the preceding is mentioned by Mr. Giles, respecting a litter of pigs, which resembled in colour a former litter by a wild boar. The best explanation of these phenomena is to suppose that connexion with the male produces a physical impression, not merely upon the ova which are ripe for impregnation, but upon others likewise that are at the time immature. In gallinaceous birds, in turkeys for instance, it is well known that a single coïtus will actually impregnate all the ova that are laid during the breeding season. The explanation which I have offered seems to me far more reasonable than any supposed influence of the imagination, the effect of which on any occasion, even in human beings, appears more than doubtful."—*Mayo's Physiology, 2d Edition,* p. 489.

*Case in which no Division existed between the Ventricles of the Heart.*—A case of this kind is related by Dr. Wittchke, in *Hufeland and Osann's Journal* for April 1828.

The patient was twenty-four years of age; had from his infancy been affected with a peculiar palpitation of the heart, unattended by any other symptom of disease. Five years ago, after an inflammation of the chest, the palpitation augmented in violence; he appeared sometimes to be in danger of suffocation. The palpitation and sense of suffocation abated when he sat upright, and pressed his breast firmly against any hard body. Medical treatment rendered him no relief: the occurrence of hemorrhoids appeared,

* Philos. Trans, 1821, p. 21.

however, in some measure to render his sufferings more tolerable. Finally, the accessions of suffocation became more violent, particularly at night: the only position in which the patient could find any ease was sitting upright. By degrees œdema of the feet showed itself, and extended to the legs; at length the abdomen became distended from effusion, and the scrotum was swollen to a great extent. His countenance was livid, swollen, and of a doughy appearance. The upper extremities presented the same phenomena.

He was first visited by Dr. W. on the 17th of November, 1825: he found him lying in bed, with his head and shoulders greatly elevated, and unable to rise. The preceding night he had suffered a very severe attack of suffocation; for fear of a return of which, he begged, with tears in his eyes, that the Doctor, by puncturing the abdomen, would relieve him of the load of water which it contained, and thus enable him to assume the erect posture. His carotids throbbed violently; the heart beat with such force that the whole of the left side of the chest was shook by its action. The motions of the chest were distinct, commencing above and extending downwards. The pulse was regular, somewhat tense, and moderately full, ninety in a minute. The respiration was rattling, as though some obstruction existed in the trachea. The patient could speak only with great difficulty; the tone of the voice was hoarse and low. The body presented all the symptoms of extensive general dropsy. Appetite and digestion not particularly affected; the tongue very red and clean; great thirst; the skin had the feel of dry parchment. The skin on the fore part of the scrotum, being irritated by the passage of the urine over it, presented an erysipelatous appearance; as also did the calves of the legs. The patient complained of considerable pain of the left thigh.

Notwithstanding the dropsy was to be viewed as an effect of disease of the heart, under which the patient evidently laboured, yet, with the view of mitigating his sufferings, as well as to take off the extreme tension of the skin, from which gangrene was to be feared, Dr. W. punctured the abdomen. Though but a small quantity of fluid was discharged, the patient felt somewhat relieved. Diuretics were directed internally; and, externally, tepid poultices, with acetate of lead and laudanum, to the left inferior extremity.

Next day, near six quarts of water discharged by the puncture; the patient much relieved; had a quiet night. Poultices exchanged for a decoction of chamomile flowers, with acetate of lead and laudanum. Seltzer water to allay the thirst.

On the ensuing day, symptoms the same. Diuretics continued.

On both of the two following days, bowels evacuated; pain of left thigh increased, with redness. Chamomile decoctions discontinued, and a decoction of oak bark, with the same additions, directed.

On the 22d of the month, dropsical symptoms considerably abated; palpitation of the heart much diminished; the patient could stand without assistance, and sat up the greater part of the day in an armchair.

On the 25th, frequent watery stools, weakness, the pulse more frequent and strong. Diuretics discontinued, and frictions to the abdomen of Spirit. Terebinth. and Camph. united by the yolk of an egg.

From this time the patient rapidly declined in strength. On the 29th gangrene of the left thigh. On the 30th, the patient died.

*Sectio cadaveris.*—The thorax only examined: it contained about a pint of bloody serum. Lungs healthy, but pressed backwards, in consequence of the augmented size of the heart; they were not greatly distended with blood, and no where adhered to the pleura. The pericardium adhered completely to the surface of the heart, with the exception of that part which is next the diaphragm, where not a trace of it was discoverable, the heart lying immediately upon the latter. The heart was at least three times its normal size, and filled with a considerable quantity of black half-coagulated blood; the parietes of both ventricles being of a thickness proportionate to the augmented bulk of the organs. The most remarkable circumstance was the absence of the septum ventriculorum, of which not a trace remained; notwithstanding which, the openings of the great vessels were entirely natural, excepting that those of the veins were somewhat enlarged; the valves perfect. The auricles, with the exception of their increased size, were also in every respect natural. The aorta arose very straight, and was filled with a fibrous coagulum. The veins were collapsed, and contained a small quantity of fluid blood; this was also the state of all the great veins of the chest. The ductus arteriosus was, as usual, entirely closed.—*North American Med. and Surg. Journal.*

---

*Influence of Operations on the Neck on the Nutrition of the Eye.*—M. MAYER has experimented for this purpose on several domestic animals. When the great sympathetic was tied in the neck, it occasioned some disorder in the nutrition of the eye on the same side, manifested by inflammation of the conjunctiva. Injury of the nervus vagus is often followed by the same effect. When both the vagus and sympathetic are tied, the effect is more manifest, and the inflammation extends to the interior of the globe of the eye.

When one carotid is tied, not much effect is produced; but, if both are secured, the eyes always suffer more or less, they lose their brightness and their vital turgescence: however, it rarely happens that complete disorganization ensues.

But, if the vagus, great sympathetic, and carotid are included in the ligature, an exudation of plastic lymph takes place on the anterior surface of the iris: this new product, which is membranous, completely closes the pupil. In time, suppuration of the cornea and staphyloma are observed.—*Bulletin.*

---

*On the Functions of different Parts of the Ear.*—Dr. CHARLES L. ESSER, in a work crowned in 1825 by the Medical Faculty of the University of Bonn, has been led, by numerous experiments, to the following conclusions:

The *cartilage of the external ear* seems to contribute little to rendering sounds more clear, but seems to augment their force not only by reflecting into the auditive canal a part of the sonorous rays, especially those that fall into the concha, but also by means of vibration which the sonorous rays produce in it, and which it transmits to the tympanum.

The *bones of the head* contribute no less than the cartilage to the propagation of sounds. This propagation does not depend solely on the nerves, as was thought by TREVIRANUS, SWAN, &c.; for a watch applied to a swelled cheek ought to produce sounds as clear through the medium of the fascial nerve as if applied on the zygomatic arch, which is not the case. The os occipitis is better fitted for propagation of sounds than the bones of the anterior parts of the head, which depends on its connexion with the labyrinth, and its nearness to the mastoidal cells. The use of these cells is not to prevent an echo in the internal ear, as M. Treviranus supposed; that function devolves solely on the eustachian tube. In many animals, the bones that surround the external ear are disposed so as to favor, in a high degree, the transmission of sounds, and they offer compensation for the absence of an external ear.

The external auditory canal is evidently the part which contributes most to the concentration and transmission of sounds to the membrana tympani.

The membrana tympani is made to vibrate by the sonorous rays that reach it: nevertheless, this is not the only use of the membrane, for the sonorous vibrations not only may reach the ear without the aid of the membrana tympani, but may even reach it with greater strength. It serves to protect the ear from external injuries.

The eustachian tube is the chief auxiliary to the membrana tympani, and fulfils four different functions.

1. It allows the air contained in the cavity of the tympanum to be in a state of equilibrium with the external air. If this equilibrium be lost, certain anomalous sensations are experienced, such as tinkling and buzzing of the ears. If the quantity of air in the cavity is augmented by strong expirations, then too strong a pressure on the membrane of the tympanum takes place, as well as on the other parts of the cavity, particularly the foramen rotundum: this pressure produces buzzing, which decreases in proportion as the atmospheric equilibrium is restored through the eustachian tube. If the air of the cavity becomes rarefied, and the eustachian tube closed by spasm, then the external air pressing on the membrane finds its way through the pores of that organ, and thus produces the tinkling of the ears.

Both these phenomena disappear as soon as the equilibrium is restored in the cavity, and this may be effected by forcing the air along the eustachian tube by breathing with the nose and mouth closed, or by introducing the end of the little finger deeply into the external meatus, and withdrawing it by degrees, making at the same time pressure against the upper surface of the canal. In this way a vacuum is formed, the membrane is brought back towards the external meatus, and the eustachian tube gives passage to the air that presses into it from the fauces. This explanation manifestly can only apply to the occasional occurrences of buzzing, &c. Chronic examples of these affections depend on cerebral congestions or anomalous actions of the nerves.

2. The second function of the eustachian tube is to admit of the occurrence of vibrations in the cavity of the tympanum, which could not be the case if it were entirely closed.

In cases of deafness occasioned by obliteration of the eustachian tube, perforation of the membrane becomes a means of cure, by re-establishing the communication with the external air.

The idea that the sides of the tube are constantly in contact with each other is incorrect.

3. The tube prevents confused vibrations of the air of the cavity, by giving them a passage outwards.

4. Finally, it serves to lead into the posterior nares the mucus secreted into the cavity and on its own surface.

The *small bones of the ear*, by means of their muscles, are capable of rendering the tympanum more or less tense; but it is difficult to conceive why, and how, this effect takes place. Their influence on the sense of hearing is not very remarkable. They serve to transmit the sonorous vibrations of the membrana tympani to the foramen ovale, although this is not their sole use.

The *labyrinth*, whose anatomical history is sufficiently understood, is still, and will probably continue to be, the obscurest part of the apparatus of hearing, as relates to its physiology. The experiments of WEBER leave it a doubtful question whether the lymph of COTUGNI exists during life, or is a production occasioned by the death of the animal.

The *vestibule*, or the membranous bags which represent it in certain animals, and the semicircular canals, appear to contribute most to the sense of hearing, but it is difficult to say in what manner. Some facts in comparative anatomy seem to show that the principal use of the semicircular canals consists in strengthening the sounds. They are larger in animals whose external ear is less favorably formed or wholly wanting. Large semicircular canals are generally accompanied with a small cochlea, and *vice versâ*.

The *cochlea* appears to be of less importance than the semicircular canals; for it soon disappears in the descending scale of animals, and birds only possess a rudiment of it. Its use appears to be, to offer a large surface to the sonorous vibrations, and to strengthen by concentrating them.

The act of distinguishing different objects is a purely intellectual function, which we must not seek for in any particular parts of an organ of sense. This power has been supposed to reside in the cochlea; but if its development was in every case to be regarded as a measure of this distinguishing power, the following order might be established: The cabiais and porcupine, whose cochlea has three turns and a half; the dog and fox, with three turns; man, the cow, the hog, and cat, with two and a half turns; the horse and the dolphin, with two and a quarter; and the hare, with two turns. Birds would occupy the lowest rank in this series. Such facts need no comments.

The part which the *acoustic nerve* plays in the sense of hearing is undoubtedly of great importance, but its manner of acting will always remain an impenetrable mystery.

The author of this interesting memoir finally arrives at the conclusion that all parts of the auditory apparatus concur in the act of sensation; but the sense of hearing itself is not explicable by means of these parts, for, like all the other senses, this is purely intellectual. The soul alone sees and hears, the rest is both blind and deaf.—*Ibid.*

---

### PATHOLOGY.

*Remarkable Case of Cataract.*—A Swedish Journal (*Arsberättelse om svenska lakare sällskapets arbetem,*) contains the following fact, communicated by Dr. WENDELSTROM.

A robust peasant, æt. sixty, who had always had excellent sight, and who had only suffered from slight gouty attacks, being occupied in cutting wood in a forest, suddenly felt that his vision was obscure. In a few hours he was completely blind, and he was obliged to be led home. He complained of no pain, nor were there any appearances of external inflammation. When he was examined by Dr. W. a few days afterwards, it was found that both eyes were affected with cataract. The operation of extraction was afterwards performed.

---

*Polypus of the Heart.*—M. RIGACCI, of Florence, mentions a case where a well-organized polypus was found after death in the heart. A young woman, affected with some disease of the heart, supposed to be an aneurismal dilatation of the left ventricle, after having been treated with digitalis and other means, died on the 18th of December, 1827. On examination of the body after death, there was found, among other things, a body of a fleshy appearance, similar to that called sarcoma, in the left ventricle of the heart. This ventricle, very much enlarged, had its walls much reduced in thickness. From the interventricular partition arose one of the roots of the morbid production. Another root took its rise from the auriculo-ventricular valve, by two peduncles. These two united, and formed a round body, two inches and two lines in length, which terminated by a ragged point, the surface of which did not appear covered by any membranous layer. On the external surface of the polypus were seen three reddish fillets, which, arising from the carneæ columnæ, extended to the morbid production, and appeared to be lost in its substance. These, examined with a good lens, were found full of reddish fluid, and were recognized as sanguiferous vessels. In order to prove the fact the more satisfactorily, two of the fillets were injected with mercury. One of them burst at the distance of an inch from the introduction of the tube, but the other was completely filled, and exhibited its divisions and ramuscules, which became lost in the substance of the polypus. The polypus, attentively dissected, was discovered to be formed of four or five fibrous strata, superposed one upon the other, and intimately united. The author remarks, that these observations do not permit a doubt that this polypus was properly organized, and that its formation took place before death.—*Antologia Firenze.*

---

*Suppression of the Urinary Secretion for more than seven Weeks, cured.*—One of the German Journals contains the history of a case of this nature, recorded by Dr. De Racum, of Riga. The subject of it was a child twelve years of age, in whom the secretion of urine was suppressed without occasioning any derangement of the other functions, or without any auxiliary or supplemental process of secretion being carried on during the period above mentioned. The introduction of a silver catheter into the bladder was not followed by the discharge of a single drop of urine. After attempting various other methods ineffectually, Dr. De Racum had recourse to the following preparation, which restored the renal secretion on the third day.

> Purified oil of Amber, two drachms; Venice Turpentine, ten drachms; Balsam Copaiva, one ounce. Thirty drops to be taken thrice a day in emulsion of almonds.

In addition to this, he directed friction with oil of turpentine twice a day on the lumbar region; and a regimen vegetable and diuretic. The author assures us that the above remedy is equally efficacious in gravel.

---

*Case of Phlegmasia Dolens; with an Account of the Morbid Appearances observed in the Iliac and Femoral Veins.* By Robert Lee, m.d. Physician-Accoucheur to the British Lying-in Hospital. (From the *Medico-Chirurgical Transactions*, vol. xv. part i.)

This case appears to us to contain more satisfactory evidence of the connexion between phlegmasia dolens and inflammation of the veins than any of those which have been previously published.

Mrs. J., æt. thirty-one, was delivered of her fifth child on the 10th of March, 1827, after a labour of twenty hours' duration, during which she frequently complained of severe pain shooting into her left thigh and leg. This pain entirely subsided subsequently to the labour, and she appeared to recover in the most favorable manner until the 14th of March, the fourth day after her confinement. She then began to experience a sense of pain in the left groin and calf of the leg, with numbness in the whole left inferior extremity; but nothing unusual could be perceived in the appearance of the limb, except a slight tumefaction in the situation of the inguinal glands, where pressure occasioned great uneasiness. She had occasional rigors. The tongue was furred, and there was much thirst; bowels open; pulse only eighty. The flow of milk and lochia natural.

March 16th, (the sixth day after parturition,) the pain of the left thigh and leg continued with increased severity, particularly from the groin to the knee, along the inner surface of the limb, where a swelling of a glistening white appearance was observed. The pulse was still eighty, and the general functions were but little deranged.

19th.—The pain had diminished, but the swelling had greatly increased, and extended to the leg and foot, which were both very tense, and did not pit on pressure. There was no discoloration of the skin. The pain of the limb was relieved by placing it in a state of moderate flexion.

21st.—The pain in the groin had abated, and the swelling appeared to decrease.

24th.—The pain of the limb was aggravated, particularly on moving it. The pulse more accelerated; skin hot and moist. She was extremely irritable and desponding.

25th, (the fifteenth day after delivery.)—When I first saw her, the whole extremity was much swollen, the intumescence being greatest in the ham and calf of the leg. The integuments wore a uniform smooth shining appearance, having a cream-like colour, and every where pitting on pressure, but more readily in some situations than in others. The temperature to the touch did not differ from that of the other limb, though she complained of a disagreeable sensation of heat throughout its whole extent, and much pain was experienced in the upper and inner part of the thigh on moving it. Immediately below Poupart's ligament, in the situation of the femoral vein, a thick, hard cord, about the size of the little finger, was distinctly felt. This cord, which rolled under the fingers, and was exquisitely sensible, could be distinctly traced three or four inches down the thigh in the course of the femoral vessels, and great pain was experienced on pressure, as low down as the middle of the thigh in the same direction. The pulsations of the femoral artery were felt in the usual situation below Poupart's ligament: pressure over this vessel excited little or no uneasiness. Pulse ninety and sharp; tongue much furred; thirst urgent; bowels confined. The lochial discharge had nearly disappeared.

Leeches were applied to the left groin and upper and inner part of the thigh: these were followed by cold lotions to the affected parts, and mild cathartics and anodynes were administered internally.

30th.—The acute pain on pressure, and motion of the limb, had subsided, and the extremity was universally œdematous. For two months after this period the limb remained so feeble as to disable her from walking, and continued larger than the other.

Eleven months after the attack the general health of the patient was restored, and she again became pregnant. On the 5th of November, 1828, she was delivered of a stillborn child, and died soon after from uterine hemorrhagy. Permission to examine the body was most reluctantly granted three days after death, and the dissection was necessarily conducted with the greatest possible despatch, from the danger of interruption on the part of the relatives.

*Appearances on dissection.*—The whole of the left inferior extremity was considerably larger than the right, but no serous fluid escaped from the incisions made through the integuments, beneath which a thick layer of peculiarly dense, granular, adipose matter was observed. The common external iliac, and femoral veins and arteries enclosed in their sheath, were removed from the body for examination. The common iliac, with its subdivisions, and the upper part of the femoral veins, so resembled a ligamentous cord, that, on opening the sheath, the vessel was not, until dissected out, distinguishable from the cellular substance surrounding it. On laying open the middle portion of the vein, a firm thin layer of ash-coloured lymph was found in some places adhering close to and uniting its sides, and in others clogging it up, but not distending it. On tracing upwards the obliterated vein, that portion which lies above Poupart's ligament was observed to become gradually smaller, so that, in the situation of the common iliac, it was lost in the surrounding cellular membrane, and no traces of its entrance into the vena cava were discernible. The vena cava itself was in its natural state. The entrance of the internal iliac was completely closed, and in the small portion of it which I had an opportunity of examining, the inner surface was coated by an adventitious membrane. The lower end of the removed vein was permeable, but its coats were much more dense than natural, and the inner coat was lined with a strong membrane, which diminished considerably its calibre, and here and there fine bands of the same substance ran from one side of the vessel to the other. The outer coat had formed strong adhesions with the artery and the common sheath. The inguinal glands adhered firmly to the veins, but were otherwise in a healthy condition.

No appearance of recent disease existed, and the density and firmness of the morbid textures evidently showed that the whole was the result of inflammation which had occurred at a remote period.

In the paper from which we have extracted the above interesting case, Dr. LEE relates other instances of phlegmasia dolens, which are interesting in a pathological point of view, and which strongly confirm the opinion he advocates, that this disease, frequently at least, consists in inflammation of the veins.

---

### PRACTICAL MEDICINE.

*Partial Palsy cured by Strychnia applied locally.*—John Greenshields, aged fifty-six, an habitual drunkard, was admitted into the Glasgow Infirmary on account of a varicose ulcer of the right leg. Ten days before, he suddenly lost the power of the left forearm and hand: the sensation of the parts remained perfect, but he was unable to take hold of any thing, or to extend the wrist and finger joints; had no headach. Being costive, his bowels were freely opened. A blister was then applied to the back of the forearm, and one eighth of a grain of strychnia sprinkled over the vesicated surface. On each successive day the application was increased, by adding the original quantity to that of the preceding day, till it amounted to one grain; after which one fourth of a grain, instead of one eighth, was to be added. From the second week he felt the parts to improve in power daily, with occasional sensation of prickling along the forearm and fingers. No obvious constitutional effect ensued. He was dismissed cured five weeks from the commencement of the treatment.

In the case of another man, with paralysis of the flexor muscles, and diminished sensation of the right leg from the knee downwards, a similar practice was pursued with the same good effect. He was dismissed cured, having been under treatment during six weeks.

At La Pitié, Dr. Bally is in the habit of treating cases of partial palsy in the above way, and is said to be very successful. In some cases he has made trial of the medicine internally, without benefit.—*Glasgow Med. Journal.*

---

*Intermittent Facial Neuralgia cured by Sulphate of Quinia, used according to the Endermic Method.*—Dr. Caucanas relates the history of this case in the *Journal Universal des Sciences Médicales* for May.

The subject was a lady, aged thirty years, who, after exposure to cold and moist air, was seized with acute pain of the left ear, which soon extended over the whole left side of the face. Various remedies were put in requisition to allay and remove this malady, which made its attack every evening. Repeated leeching the part, opiates, the carbonate of iron, were all tried in vain. During the administration of this last-mentioned article, the stomach became excessively irritable. The periodicity of the disease seemed to indicate the use of the quinia, which could not, however, be given in the usual manner, on account of the state of the stomach. On a vesicated surface on the arm, therefore, fifteen grains of the sulphate of quinia, mixed with some cerate, were applied. The day was passed tranquilly; no paroxysm during the night, and seven hours of sleep obtained. A similar application on the following day gave a night of entire tranquillity. Two more days of treatment were sufficient for an entire cure.

---

*Polydipsia cured by Camphor.*—Dr. Allert, of Bromberg, relates an instance of excessive thirst which occurred in a female. Notwithstanding the incredible quantity of cold water drank by the patient, the thirst was not in the least abated. Her tongue was red, and her feet began to exhibit appearances of œdema. The cause of the affection could not be determined. After the employment of many ineffectual remedies, the patient was finally, speedily, and fully cured by the exhibition of large doses of camphor.—*Journal der Practischen Heilkunde.*

---

*Iodine in Mammary Tumors.*—The August Number of the *Revue Medicale* opens with a paper on the above subject, by Dr. Bayle.

In the first case, of a suppurated tumor of the mamma of long standing, a drachm per day of an ointment into which there entered six grains of the hydriodate of potass was prescribed, and the patient took also fifteen drops of the tincture of iodine three times a day. At the expiration of fifteen days there was an evident amelioration. An uneasy dragging pain of the stomach indicated the necessity of desisting from the use of the tincture. The quantity of the hydriodate was progressively augmented, so that finally an ounce of the ointment contained two drachms of the salt. The tincture was prescribed, and occasionally discontinued, according to its toleration by the stomach. Applied by friction to the tumor itself, the tincture caused, three or four times, a general inflammation of the breast, requiring the temporary suspension of its use, and a recourse to emollients; but it was observable that, after each of these accidents, there was a more decided progress of resolution. This treatment, persevered in for three months and a half, was productive of entire relief and a disappearance of all glandular engorgement. The general health was at the same time much improved, and the menses, which had been suspended for eighteen months, were restored.

The second case was of an indolent tumor of the breast, of a scrofulous appearance, of two years' duration, and which, rebellious to the common methods of treatment of scrofula, was cured by the hydriodate of potass. A scruple of the ointment of the salt, introduced under the axilla every night for six weeks, sufficed for this purpose.

The third case was of eighteen years' duration. It was marked by an induration of two inches extent round the nipple, the consequence of a wound of that part, and accompanied by flying pains. For three months before beginning with the iodine, there had been an increase of the tumor by a fall: it was hard, bilobular, and the seat of frequent and lancinating pains. A resolution of the indurated gland was accomplished by means of frictions of the hydriodate of potass, and the tincture of the iodine internally.

In this case it was found that the excessive sensibility of the stomach, by which the patient was unable to retain a common purge, subsided under the influence of the vinous tincture of opium. She was thus enabled to take, without interruption, the tincture of iodine, six drops three times a day, gradually increasing the dose. The omission of the laudanum for a single day caused the stomach immediately to reject the iodine.

In the fourth case, distinguished by a cancerous and fetid ulcer of the breast, with cachexia, there had been an amelioration of the symptoms produced by Fowler's solution, and a return of all the bad symptoms by the use of compression.

The administration of the iodine by tincture internally, and by ointment of the hydriodate of potass externally, was followed by a rapid amendment, and nearly complete cicatrization of the ulcer. The general health was also much improved. Finally, however, there was a relapse, followed by death.

---

*On the Use of Iodine in several Diseases.* By Wm. M. Fahnestock, m.d.

Case I.—Mary, ætat. twenty-eight, a sister of the Antietam nunnery of Franklin county in this state, discovered the enlargement of the thyroid gland six years since, which continued to enlarge until it had obtained the size of a walnut, and was hard and painful. The mild tincture was prescribed in the ordinary doses, and the Liquor Potassæ Hydriodatis ordered to be used externally. The tumor at present (having used the medicine seven weeks,) is scarcely perceptible, soft, and free of pain or any uneasy sensation.

We have now five other cases under an equally successful treatment.

Case II.—Perplexed with a case obscure in its nature, and very concealed in its operations, which appeared to be a periodical congestion of the whole glandular system, associated with derangement of the catamenia, in a young lady of nineteen, and recollecting the encomiums of Coindet and Coster* on the iodine as an emmenagogue, and its usefulness in scrofulous affections, I recurred to it with the most signal advantage. About the usual period of menstruating, the superficial glands became enlarged and painful, sometimes highly inflamed, and occasionally producing imperfect suppuration, accompanied by nausea, indigestion, and eructations, which evidenced a disordered state of the mesenteric system. The discharge from the uterus was but

* Archives Générales de Médecine.

small, and attended by much pain, and frequently scarcely any show at all. The tincture was administered in the syrup of sarsaparilla, and was continued three months, with the happiest effects, and seemingly with permanent benefit. There are no cases which come under the care of the physician more complicated and interesting than the derangements of the uterine system, or for the relief of which the young aspirant gains more of the applause and confidence of the influential part of the community. It therefore affords the most fertile field for anxious but cautious and prudent experiment. I have also the pleasure of adding my testimony to that of M. Gimele,* of its usefulness in chronic leucorrhœa, from a case now under treatment, but not sufficiently recovered to report as perfectly relieved.

Case III.—Another formidable disease, which has combated every remedy and defied the surgeon's knife, may be found to yield to this very active agent, fungus hæmatodes. An incipient case came under our observation, in which the limb had not yet attained very great enlargement, but was knotted, and bore all the characteristics of the genuine fungus hæmatodes: it yielded most effectually to the tincture and unguentum.

We have never found any of the bad effects arise from its use as exhibited in the lamentable picture of the English author upon this substance. A little sickness of the stomach occurred in the first case, which is to be attributed to the strength of Magendie's tincture; and a little vertigo attended the second, probably from increasing the dose too rapidly; both of which were relieved by omitting the remedy for one day. But, with the mild tincture, we have never witnessed any unpleasant symptom; and it may be proper to add, that Decarro, Coindet, Erlinger, Formey, and a host of others, who have experimented most extensively, never complained of any injurious qualities. It is well, however, to observe, that the *burnt sponge*, which has been used as the same, has not precisely the same operation, and contains some deleterious properties, which have been particularly remarked by Hufeland,† as being noxious to persons disposed to phthisis and spitting of blood; and it is doubted by Foderé and M. Hetch whether it contains any iodine whatever. Much may depend on the quality of the tinctures, as it has been ascertained that, when suffered to stand any time, it deposits crystals, and may form the ioduretted hydriodic acid: therefore it should always be prepared for immediate use, and in small quantities. These precautions may obviate the evils complained of so bitterly; to which may be added, premising its exhibition by evacuating the alimentary canal, and occasionally giving a gentle cathartic, as calcined magnesia, should any untoward symptoms occur.—*American Journal of Med. Sciences.*

---

*Cancer of the Uterus cured by Injections of Hydrocyanic Acid.*—A case of this nature was reported by Dr. Bruni to the Medico-Physical Society of Florence, at one of its sittings in March. The injections were made four times a day. The acid was prepared agreeably to the process of Scheele, and four denarii were mixed with four pints of barley water. Cicuta and aloes were administered internally. During the first few days, the injections caused sharp cutting pains of the severest kind; but the patient having passed by the vulva fragments of a membranous and fleshy substance, her pains be-

* Revue Médicale.

† Hufeland and Osann's Journal der Pract. Heilkunde, Feb. 1828.

came from that time less severe: she regained her strength and flesh to such a degree, that in six months there was not a vestige of disease of the uterus. The menses returned at regular intervals.

### SURGERY.

*Cancer of the Penis from Phymosis.*—M. Roux lately observed, at La Charité, that phymosis is one of the most frequent causes of cancer of the penis. All the patients upon whom he had operated for cancer of that organ had either congenital or accidental phymosis. When the prepuce is in this state, it almost always becomes inflamed and hardened, and sometimes cancerous. Independent of the sebaceous matter that collects around the neck of the glans, and which frequently produces ulceration, in sexual intercourse the skin of the prepuce is distended and often torn; and, even if cicatrization does take place, the part is again torn. M. Roux also referred to an English surgeon of high authority, who is of the same opinion. In twelve amputations of the penis which he had performed, nine had been rendered necessary by cancer of the prepuce and glans, the consequence of phymosis. M. R., therefore, recommends that those persons who have phymosis should be relieved as soon as possible.

*Seton successfully applied in disunited Fracture.* By Dr. Dohldorf. (*La Lancette Française.*)

Mr. Amesbury says, that, although he has heard of many cases of non-union treated by the employment of setons, he is not aware that there are more than three treated in this country, where its operation appears to have brought about consolidation of the bone.* The following case is, therefore, interesting.

J. R., twenty-four years old, of good constitution, broke his leg in January 1826, and was attended for ten weeks by a country surgeon. At this time the fracture was not united, and the ends of the bone were not in apposition. Many plans of treatment were tried without success, and in September he was placed under the care of Dr. Dohldorf. The left thigh was not lessened in bulk, but it was rather shorter than the right. When the patient stood up, the limb was turned inwards. The fracture was about the middle of the bone. The broken extremities were not enlarged, and were perfectly free from any adhesion; for the lower part of the limb could be moved in any direction without difficulty or pain. General health good. The application of a seton between the fractured extremities of the bone was determined upon. The operation was performed on October 3d. The inflammation which followed was so severe that it was necessary to withdraw the seton eight days after. Free suppuration took place, and several fistulous abscesses formed between the muscles. But little hopes of benefit from the operation were now entertained, and the life of the patient was not considered altogether safe. At the expiration of a month, however, the fracture began to consolidate. The wound made for the introduction of the seton, and the abscesses, healed. Towards the end of December very solid callus was formed; and, to prevent excessive ossific deposition, which took place too rapidly, pressure was applied. At the beginning of May the length of the two legs was equal, and the patient complained of nothing more than very slight weakness in the left limb.

* Observations on the Nature and Treatment of Fractures, 1828, p. 225.

*Aneurism of the Brachial Artery cured by Pressure.*—M. Tetu, a regimental surgeon, details the particulars of a case of false aneurism at the bend of the arm, cured by pressure, which method he prefers to the ligature now so universally recommended. His patient in twenty-one days was cured; the tumor having disappeared, and the artery being obliterated for four inches. This is the second case in which he succeeded: the first patient recovered in sixteen days. His mode of compression is by a bandage methodically applied from the hand, by a graduated compress over the tumor and in course of the artery, and covered by a splint two inches wide and fifteen in length. Another splint, four inches wide and also fifteen long, was applied on the opposite side of the arm. Both were secured by a bandage, so as partially to interrupt the blood in the injured artery. In a few days the pressure was increased, so as to arrest the passage of blood in the vessel: the circulation, however, continued in the anastomosing vessels, owing to the arrangement of the splints, by which they were protected from compression.—*Journal Universelle.*

---

M. Civiale's *Operation of breaking down the Stone in the Bladder.* (Extract from La Roche's "Account of Surgical Operations in Paris," *North American Med. and Surg. Journal.*)

The cases in which I saw M. Civiale operate were all very interesting, and the results reflect great credit on him. The first was that of a man on whom M. Dupuytren had attempted the same method, but failed. The patient, being thought too much enfeebled by disease to bear the operation of lithotomy, was sent to the country to recruit a little strength. After returning to the city, instead of going once more to the Hôtel Dieu, he entered the Pitié, was sounded by M. Lisfranc, experienced an inflammation of his testicles, and, after recovering from this, was placed by M. L. in charge of M. Civiale. The first operation by this surgeon was performed at the hospital, in presence of a large class of students and of several surgeons, both foreign and native. M. C. experienced none of the difficulty which M. Dupuytren had done; he introduced a larger instrument into the bladder, seized the stone, and ground it. All this was done in about as little time as I employ in stating it. The stone proved to be very brittle, several fragments were destroyed, and nearly a teaspoonful of sand was passed with the urine immediately after the operation, and during the course of the day. As the man's constitution is very feeble, (in fact, as he is a bad subject for any operation, however trifling,) he was soon sent to bed. Experiencing no unpleasant effects from this first trial, he very gladly submitted to a second a few days after. The stone was again bored, and several other fragments destroyed. Since then, his bladder having experienced a slight degree of irritation, and, after this had subsided, one of his testicles having swelled, the operation has not been repeated. It is thought that a few days will suffice to set every thing to rights, when a third trial will be made. I have little doubt that in a few weeks this man will be completely rid of his stone.*

I attended at M. C.'s house on several occasions, and saw him there perform his operation on two or three individuals. One of the individuals, a man of about sixty-five or seventy years of age, had submitted twice to the

* One of the students informed me today (Sept. 8,) that the man was quite well, and that the operation would be repeated in a few days.

operation of lithotomy: once over the pubis, and the other time by the perineum. The consequence is that his bladder had two cicatrices, one above and the other below, which rendered it very irregular. Owing to this, M. C. experiences, at each repetition of his operation, considerable difficulty in finding the stones, and particularly the small fragments, and the patient suffers a good deal more than others generally do. Yet the former always succeeds; and the latter gets up laughing and joking, dresses himself, and walks off as if nothing was the matter with him, always stating that he will soon return to have the business completed, and that he is sorry he did not know of this method before; for, had he done so, he would not have submitted to the others. Can the advantage of this method be indicated more satisfactorily than by such facts?*

Another patient, also far advanced in years, has the opening of the urethra far below the head of the penis. This occasioned some difficulty in introducing the instruments. But M. C. finally succeeded; seized the stone, and with more than usual difficulty ground a good portion of it; for it proved as hard as marble. This man also walked home very quietly, and experienced comparatively little pain, except on the first introduction of the instruments. Since writing the above I have assisted, in company with our friends, Dr. BROWN and Mr. RALSTON, at the second operation on this patient. He bore it much better than on the former occasion, complained of less pain, and walked off in good spirits, blessing M. Civiale with much warmth. A few minutes after I met him on the Boulevard. He told me that he had no pain, which I could easily perceive, as he walked as erect, and with as cheerful a countenance, as if nothing had occurred. Mr. Ralston, who is no medical man, and consequently must be admitted to be a disinterested and impartial judge, will bear testimony of the ease with which the operation is performed, at least by M. Civiale.

In terminating what I have to say of M. Civiale and his instruments, I must not omit mentioning that, whilst at Pavia, my friend, Professor RIGONI, informed me that the celebrated SCARPA had formerly publicly expressed a very unfavorable opinion of the operation, an opinion formed merely from examining a set of instruments made at Milan. In the spring of 1827, however, M. Civiale, who had gone to Genoa on business, crossed over to Pavia for the express purpose of seeing Scarpa. No sooner had this remarkable man seen M. Civiale's instruments, and been shown the mechanism of the operator, than he perceived that the unfavorable opinion he had formed of this method was attributable to the imperfection of the Milanese instruments above alluded to, and not to the method itself; and candidly and honourably retracted his former judgment. Scarpa has not himself written any thing relatively to this change of sentiment; but requested Professor Rigoni to do so for him, in a letter to Professor Pazzini, of Lucca. This letter is published in one of the Numbers of Omodei's Annals, and ought to be read and meditated upon by every inexperienced opponent of the *grinding* method. They will, I hope,

* From comparative tables made at Paris, it appears that one out of five patients die from the operation of lithotomy. In 150 cases in which Civiale's method has been employed, not more than five or six have ended fatally; and it must be observed that most of these occurred in the practice of surgeons who were inexperienced in the use of the instruments for grinding and breaking the stone.

allow that Scarpa's judgment in surgical matters is of as much weight as theirs; and, assuredly, as if he thinks himself authorised, after examining the thing for himself, and after mature reflection, to express a decidedly favorable opinion relatively to M. Civiale's instruments, I may be allowed to think that they will not be disgraced by looking a little into the matter; or, at least, by expressing themselves in a less decided tone against a surgical point, of which they have not the least knowledge, and on which, consequently, they are not able to pronounce.—*North American Med. and Surg. Journal.*

---

### MATERIA MEDICA.

*Ergot of Rye.*—In the *Bulletin des Sciences Médicales* for July, we find a notice of a work published last year by M. Courhaut, health-officer at Chalons, and medical practitioner in the departments of Saone-et-Loire and of Allier, which contains some interesting particulars respecting the effects of ergotic rye, when used in quantity as aliment by the inhabitants. During what the author calls an *ergotic year*, when rye in which ergot predominates is eaten, the individuals so using it suffer from gangrene of the limbs. There is at first a tingling sensation in the part, which assumes a roseate hue; the pulse is gradually weaker, and finally ceases to beat; then follows coldness, swelling, violaceous colour, sphacelus, and a separation of the limb or a portion of it.

Ergotic bread used by nurses for four or five days dries up the secretion of milk. It causes abortion after ten or fifteen days' use of it.

These facts are the more valuable as the result of extensive observation, and not pressed in support of any particular hypothesis. It is well to remark that the largest administration of the ergot as a medicine cannot equal what is taken in the ergotic bread, and in which the baneful agent is nearly an eighth part of the whole mass. By such an estimate, one to two ounces of ergot must be ingested daily.

The chief preventive and curative agent in the hands of M. Courhaut, after abstinence from the ergotic bread, is ammonia, rubbed on the affected parts, and taken internally with bark. He asserts that he has had 300 persons variously affected with ergotism under his care, and has only lost one.—*Ibid.*

---

### MEDICAL JURISPRUDENCE.

*Asphyxia.*—M. Leroy, in a memoir presented to the Royal Academy of Medicine in 1826, had pointed out the dangerous consequences of pulmonary insufflation; a means so commonly had recourse to in order to restore vitality to drowned persons. He showed that it was sufficient for air to be driven once with force into the chest of rabbits and sheep to produce in them instant death. In a new work, M. Leroy, by still more numerous and diversified experiments, endeavours to prove that pulmonary insufflation, so far from being entitled to the first place in the treatment of asphyxia, ought only to be practised in particular cases, and with great circumspection.

The principal facts resulting from the experiments of M. Leroy are the following:

The dangers of insufflation are in the inverse ratio of the resistance and density of the lungs of the animal. Thus the specific gravity of the lungs of a dog is double that of a sheep, and accordingly insufflation, almost invariably fatal to the latter, only produces in the dog a great difficulty of respiration, and rarely death. The density of the lungs of the new-born child is greater than that of the adult; and hence we may infer that pulmonary insufflation would be less dangerous to the infant, and such, according to the experiments of M. Leroy, is the fact.

Death from sudden insufflation depends on various disorders. There is almost always a rupture of the pulmonary cells, the air is effused into the cavity of the chest, compresses the lungs, which are shrunk, and prevents respiration. When death after sudden insufflation is not accompanied by effusion in the chest, air is found in the blood-vessels, and ecchymosis on the surface of the lungs.

M. Leroy, in accordance with the opinion of DESGRANGES, thinks that asphyxia by submersion is the direct effect of syncope, and that the greater number of drowned persons recalled to life after a certain interval had been seized with fainting at the moment of immersion, so that the circulation was stopped at the same time with respiration, and dark blood did not flow into the arteries. That the inference from this belief was not acted on, is a matter of astonishment to M. Leroy. The treatment for syncope is that which ought to be put in use in cases of asphyxia from submersion. Galvanism over the diaphragm by means of fine needles, has been proposed by the author; but this demands some time and preparation, and can only be done by a physician. M. Leroy has, since he proposed this plan, tried with advantage the practice of simple pressure on the thorax and abdomen: the ribs and diaphragm, thrown back by this manœuvre, return on themselves in virtue of their elasticity, and cause an enlargement of the chest, and consequently the entrance of air, which may be expelled by renewed pressure. These movements make an artificial respiration.—*Ibid.*

### NATURAL HISTORY.

*Vegetation in Air at different Pressures.*—M. DOBEREINER took two equal glass vessels of 320 cubic inches capacity each: in these were put portions of the same earth in which two portions of barley had been sown, and moistened to the same degree. The air was now exhausted from one vessel until the pressure equalled fourteen inches of mercury, and in the other it was condensed until the pressure equalled fifty-six inches. Germination took place in equal times, and the leaflets were equally green; but, at the end of fifteen days, the shoots in the rarefied air were only six inches long, but in the condensed air from nine to ten inches. The former were expanded and soft, the latter rolled round the stem and solid: the former were wet on their surface, and especially at the extremities; the latter nearly dry. "I am disposed (says M. Dobereiner,) to believe that the diminution in the size of plants, as they rise on mountains into higher regions, depends more on the diminution of pressure than of heat." The phenomenon of drops of water on the leaves in rarefied air calls to mind the relation of a young Englishman, who, whilst passing through Spanish America as a prisoner, remarked that "on the highest mountains of the country the trees continually transpired a quantity of water, even in the driest weather, the water falling sometimes like rain." *Bib. Universelle.*

*Native Sulphuric Acid.*—Professor EATON has described the natural occurrence of sulphuric acid in large quantities, both in a diluted and a concentrated state, in the town of Byron, Genessee county, ten miles south of the

Erie canal. The place has been known in the vicinity, for seventeen years, by the name of the *sour springs*. The place consists of a hillock 230 feet long and 100 broad, elevated about five feet above the surrounding plain: its greatest extent is north and south; it consists of an ash-coloured alluvion, containing an immense quantity of exceedingly minute grains of iron pyrites. It is mostly covered with a coat of charred vegetable matter, four or five inches thick, and black as charcoal; the same kind of matter extends on all sides, from the base of the hillock over the plain. Its charred side is caused wholly by the sulphuric acid. Several holes have been dug in the hill, which now contain turbid dilute sulphuric acid; also the depressions on the meadow ground around it. The strength of the acid increases in a time of drought. When Professor Eaton examined it, much rain had recently fallen, and the acid was very dilute in most places, but it was strong in some, and appeared to be quite concentrated and nearly dry in the charred vegetable coat. In this state it was diffused through the whole piece of ground, which presented the charred appearance to the depth of twelve or fifteen inches, and in some places three or four feet. But it was every where the strongest at the surface.

In wet spring seasons it appeared that adders-tongue, and some other plants, flowered on this hillock sooner than on the adjoining grounds; but, as soon as the spring rains began to decline, then the vegetables withered away, and appeared as if scorched.

About two miles east of this place is another sulphuric acid spring, still more remarkable in one respect. The quantity of water from the spring is in sufficient quantity to turn a light grist mill, and yet there is so much sulphuric acid present in it, that the stream will constantly redden violets, and its water coagulate milk. Several other sour springs were mentioned as existing in the neighbourhood.

It is supposed that the sulphuric acid is prodnced in some way by the de. composition of the pyrites in the soil.—*Silliman's Journal.*

---

*Spontaneous Human Combustion.*—That cases happen in which the human being, even when alive, undergoes a sudden destruction, as if by a consuming process, cannot be doubted; and these are now so numerous as to have induced M. Julia de Fontenelle to read a paper on the subject to the Academy of Sciences of Paris. Fifteen instances are particularly described by him, from the details of which the following general results are obtained:

1. Generally those who have died by spontaneous combustion have indulged in excess of alcoholic liquors.

2. The combustion is almost always general, but in some cases may be partial.

3. It is rare amongst men: the women have, in almost every case, been aged.

4. The body and the viscera have always been burnt; whilst the feet, hands, and top of the head, have almost always been preserved.

5. Although it is known by experience that a very large quantity of wood is required to burn a corpse, this particular kind of incineration occurs without inflaming the most combustible substances of an ordinary kind near it.

6. It has not been shown in any case that the presence of an inflamed body is necessary to commence this kind of combustion.

7. Water, instead of extinguishing the flame, appears to give it more activity; and, when the flame has disappeared, the combustion proceeds within.

8. They occur more frequently in winter than in summer.

9. The cure of general combustion has never been effected, only of partial ones.

10. Those to whom it has happened have experienced a sensation of strong internal heat.—

11. It is suddenly developed, and consumes the body in a few hours.

12. Those parts which are not reached by the fire are affected by sphacele.

13. A putrid degeneration ensues, which causes gangrene.

14. The residue of this combustion is composed of greasy cinders and an unctuous fatty matter, both having a fetid odour, which is perceived at a great distance.

These results show the inapplicability of the explanations which have sometimes been given of this effect. Considering how much fuel is required to consume a corpse, it is impossible to suppose that, in these cases, the combustion is occasioned by the inflammable nature of alcohol, or of hydrogen, which may be supposed to be evolved; and, again, the ordinary products of the combustion of animal matter is a very spongy, black, shining charcoal, fetid, and incinerating only at a very high temperature; whilst, in spontaneous human combustion, the temperature produced is so low as not to inflame neighbouring bodies.

M. Julia de Fontenelle, therefore, considers this case of combustion to depend upon a very advanced and putrid degeneration of the system, which suddenly produces very combustible substances, at the expense of the muscular fibre, &c.; and, these inflaming spontaneously, (by means of the opposite electricities, &c.) cause the resulting effect. This degeneration is considered as presenting a perfect analogy with vegetable putrid fermentation and putrefaction. The putrefaction of vegetables is known to occasion the development of so much heat as sometimes to cause their inflammation.

These human combustions do not depend upon the combination of the oxygen of the atmosphere: because, 1st, there is not sufficient heat evolved; 2d, there is not the production of a charcoal requiring a high heat for its incineration; 3d, there are no ammoniacal products formed. The effects appear to depend altogether upon a new arrangement of the elements previously existing in the human body.—*Quarterly Journal of Science*, from the *Bull. Univ.*

